# Hapsolutely: a user-friendly tool integrating haplotype phasing, network construction, and haploweb calculation

**DOI:** 10.1093/bioadv/vbae083

**Published:** 2024-06-05

**Authors:** Miguel Vences, Stefanos Patmanidis, Jan-Christopher Schmidt, Michael Matschiner, Aurélien Miralles, Susanne S Renner

**Affiliations:** Division of Evolutionary Biology, Zoological Institute, Technische Universität Braunschweig, 38106 Braunschweig, Germany; Department of Computer Science, School of Electrical and Computer Engineering, National Technical University of Athens, 15780 Athens, Greece; Division of Evolutionary Biology, Zoological Institute, Technische Universität Braunschweig, 38106 Braunschweig, Germany; Natural History Museum, University of Oslo, 0562 Oslo, Norway; Division of Evolutionary Biology, Zoological Institute, Technische Universität Braunschweig, 38106 Braunschweig, Germany; Institut de Systématique, Évolution, Biodiversité (ISYEB), Muséum National d’Histoire Naturelle, CNRS, Sorbonne Université, EPHE, 75005 Paris, France; Department of Biology, Washington University, Saint Louis, MO 63130, United States

## Abstract

**Motivation:**

Haplotype networks are a routine approach to visualize relationships among alleles. Such visual analysis of single-locus data is still of importance, especially in species diagnosis and delimitation, where a limited amount of sequence data usually are available and sufficient, along with other datasets in the framework of integrative taxonomy. In diploid organisms, this often requires separating (phasing) sequences with heterozygotic positions, and typically separate programs are required for phasing, reformatting of input files, and haplotype network construction. We therefore developed Hapsolutely, a user-friendly program with an ergonomic graphical user interface that integrates haplotype phasing from single-locus sequences with five approaches for network/genealogy reconstruction.

**Results:**

Among the novel options implemented, Hapsolutely integrates phasing and graphical reconstruction steps of haplotype networks, supports input of species partition data in the common SPART and SPART-XML formats, and calculates and visualizes haplowebs and fields for recombination, thus allowing graphical comparison of allele distribution and allele sharing among subsets for the purpose of species delimitation. The new tool has been specifically developed with a focus on the workflow in alpha-taxonomy, where exploring fields for recombination across alternative species partitions may help species delimitation.

**Availability and implementation:**

Hapsolutely is written in Python, and integrates code from Phase, SeqPHASE, and PopART in C++ and Haxe. Compiled stand-alone executables for MS Windows and Mac OS along with a detailed manual can be downloaded from https://www.itaxotools.org; the source code is openly available on GitHub (https://github.com/iTaxoTools/Hapsolutely).

## Introduction

Inferring the genealogical relationships among haplotypes—sets of spatially proximate DNA variations that tend to be inherited together—is an important component of studying demographic, phylogeographic, and population-genetic processes (Bossart and Prowell 1998, Emerson *et al.* 2001, Paradis 2018). In the case of diploid individuals, haplotype analyses typically require separating alleles from two parents via computational haplotype phasing (Stephens *et al.* 2001, Browning and Browning 2011) For single-locus datasets, haplotype relationships are often represented as networks that can take into account multifurcations (Posada and Crandall 2001) and that show the number of mutational steps between unique haplotypes and their frequency in the studied populations. Numerous methods have been proposed to reconstruct such haplotype networks and haplotype genealogies: directly from DNA sequences based on statistical parsimony (Templeton *et al.* 1992), maximum parsimony (Branders and Mardulyn 2016), median-joining (Bandelt *et al.* 1999), minimum cost arborescence (Li *et al*. 2023); or via distances based on a minimum spanning tree (Kruskal 1956), minimum spanning network (Bandelt *et al.* 1999), integer neighbor-joining (Leigh and Bryant 2015), randomized minimum spanning tree (Paradis 2018), or the Fitch algorithm (Matschiner 2016).

With the rise of high-throughput sequencing, haplotype analysis has shifted from the analysis and visualization of single-locus networks to chromosome-scale haplotype reconstruction (Garg 2021). It also now includes applications in fields such as haplotype-based genome-wide association studies (Bhat *et al.* 2021) and more abstract visual representation of variant profiles (e.g. Farrer 2021). However, single-locus haplotype networks are still being extensively used, for instance, to illustrate relationships among SARS-CoV-2 genomes (e.g. Mostefai *et al.* 2022). In the field of biological taxonomy, DNA barcode genes can be useful to generate initial (primary) species hypotheses (e.g. Puillandre *et al.* 2012), and haplotype networks can then be used for testing these primary species hypotheses by inferring haplotype sharing (HS) in unlinked nuclear-encoded markers among the subsets (e.g. Lin *et al.* 2018, Petzold and Hassanin 2020, Jamdade *et al.* 2022).

Another explicit species delimitation approach based on haplotypes from diploid organisms is the reconstruction of “fields for recombination” (FFR), i.e. groups of individuals with mutual allelic exclusivity (Doyle 1995), which for single-locus data can be visualized as so-called haplowebs (Flot *et al.* 2010). The conceptual background for this approach is derived from the genealogical concordance species criterion (Avise and Wollenberg 1997) with absence of allele sharing in multiple unlinked markers indicating that the respective subsets probably represent independent evolutionary lineages.

## Scope

Available software tools for haplotype network reconstruction are TCS (Clement *et al.* 2000), Network (http://www.fluxus-engineering.com), Arlequin (Excoffier and Lischer 2010), Fitchi (Matschiner 2016), the R package pegas (Paradis 2018), HaplowebMaker (Spöri and Flot 2020), and PopART (Leigh and Bryant 2015) (Table 1). Especially PopART is a highly versatile, user-friendly program driven by a graphical user interface (GUI). Haplotype phasing from single-locus data with the original Phase program (Stephens *et al.* 2001), however, is a convoluted process that requires interconverting input and output files with SeqPHASE (Flot 2010) or the use of DnaSP which implements phasing from Fasta files (Librado and Rozas 2009). So far, no standalone program exists that directly couples phasing with network visualization.

**Table 1.** Comparison of programs for sequence phasing and calculation of haplotype networks and haplotype genealogies.

Program	Language	Operating systems	GUI	Phasing	Sequence input	Network algorithms	Interactive network editing	Extras	Comments
Hapsolutely	Python (wraps C++ and Haxe components)	Win, Mac, Linux	Yes	Yes	Fasta, MolD-Fasta, tsv	TCS, TSW, MSN, MJN, FTN	Yes	Species partitions from SPART; visualizes fields for recombination	Integrates code from Phase, SeqPHASE, Fitchi and PopART
PopART	C++	Win, Mac, Linux	Yes	No	Nexus	TCS, TSW, MSN, MJN, AMP, INJ	Yes	Geographical plotting of network; sequence statistics	
TCS	Java	Win, Mac (Linux)	Yes	No	Nexus, Phylip	TCS	Yes		
Network	Unknown (not open source)	Win	Yes	No	Fasta	MJN, RMN	Yes	Extensive functions for data editing, complexity reduction, weighing	
HaplowebMaker	Java/Haxe	Webtool	Yes	No	Fasta	MJN	No	Can deal simultaneously with input files from various markers	Webserver software primarily for calculation of fields for recombination
Fitchi	Python	(Python)	No	No	Nexus	FTN	No		Requires calculation of phylogenetic tree from external program
HapView (Haplotype Viewer, HaploView)	Java	Windows, Mac, Linux	Yes	No	Fasta*	FTN	Yes		Requires calculation of phylogenetic tree from external program
pegas	R	(R)	No	No	Fasta and others	MSN, MJN	No		
SeqPHASE	Perl/Python/Java/Haxe	Webtool	(Yes)	NA	Fasta	NA	NA		Tool for interconverting Phase input/output file formats
DnaSP	Visual Basic	Win, Mac, Linus	Yes	Yes	Fasta	NA	NA	Performs numerous other population genetic calculations.	Implements the original Phase algorithm

AMP, ancestral maximum parsimony; FTG, Fitch genealogy; INJ, integer neighbor-joining; MJN, median joining network; MSN; minimum spanning network; RMN, reduced median network; TCS, Templeton, Crandall and Sing network (statistical parsimony); TSW; tight span walker.

**Figure 1.**
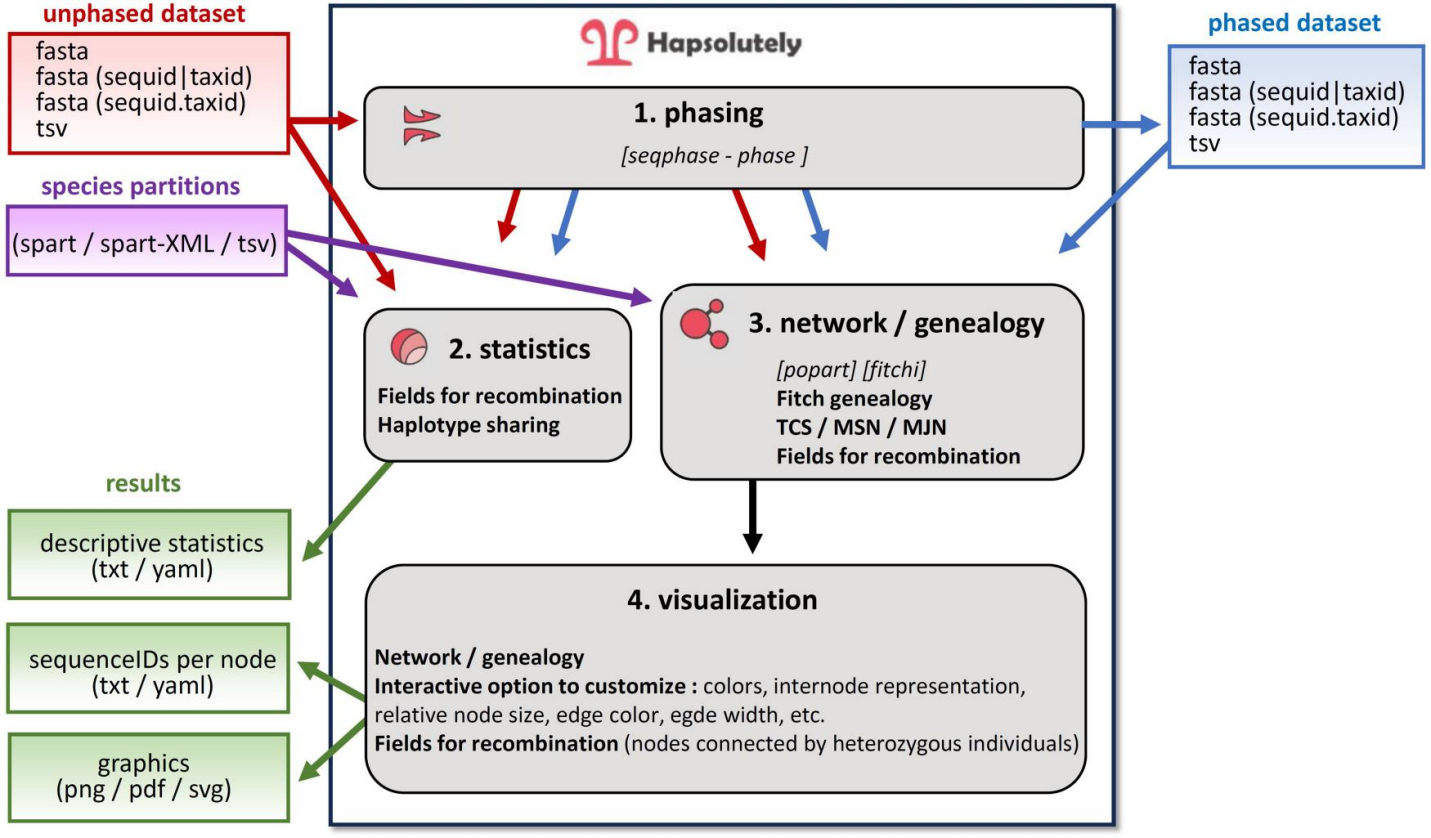
Work flow of Hapsolutely. The graph summarizes the various steps and functions of the programs, as well as input and output files produced. For details, see the manual of the program (available at https://github.com/iTaxoTools/Hapsolutely).

We here present an integrated tool, Hapsolutely, developed for the iTaxoTools project (Vences *et al*. 2021), to facilitate the tasks of haplotype phasing and haplotype network reconstruction from single-locus sequence data (Fig. 1). The program has an emphasis on user-friendliness and on functions useful for species delimitation, such as haploweb visualization and SPART (species partition) format support (Flot *et al.* 2010, Miralles *et al.* 2021). Hapsolutely is provided as compiled GUI-driven standalone executable for Windows and Mac systems, with the original code being available from Github.

## Implementation

The phasing step of Hapsolutely (also available as separate tool ConvPhase, with its name derived from “convenient phasing”; see Data availability below) wraps the original code of Phase (Stephens *et al.* 2001), along with that of SeqPHASE (Flot 2010), extended with options for a variety of input and output file formats. It accepts input in FASTA format, and can automatically recognize taxon identifiers from the sequence name when included. Data tables (as tab-delimited text) are also accepted. Diploid sequences can then be phased, with several parameters adjustable via the GUI, and output is provided in the user-specified format. The two-phased haploid sequences derived from each initial diploid sequence are denoted with an “a” and “b” separated from the individual identifier by an underscore, allowing the straightforward use of the output file in programs such as MOLD for molecular diagnosis (Fedosov *et al.* 2022), or for reconstructing a network in the program HapView (Table 1) if desired. In table format, the allele modifiers are provided as separate column for further curation in spreadsheet editors.

The integrated workflow of Hapsolutely only requires a few clicks to produce customizable and publication-ready haplotype network graphics, starting from unphased FASTA sequence alignments: (i) the program accepts unphased or phased sequences as input and in the former case, performs the phasing, and (ii) then uses the phased sequences to reconstruct haplotype networks (Fig. 1). The program also accepts sequences of haploid organellar markers, such as mitochondrial DNA or bacterial or viral markers, for which networks can be reconstructed without phasing.

For the network construction step, the following options are available:

- Median joining, minimum spanning, and statistical parsimony (TCS) network reconstruction, making use of the respective algorithms from PopART (Leigh and Bryant 2015).
- Haplotype genealogies reconstructed with the Fitch algorithm, from an uploaded user tree (ideally a maximum likelihood tree) or from a newly calculated maximum parsimony tree, and subsequent execution of the Fitchi code (Matschiner 2016).

**Figure 2.**
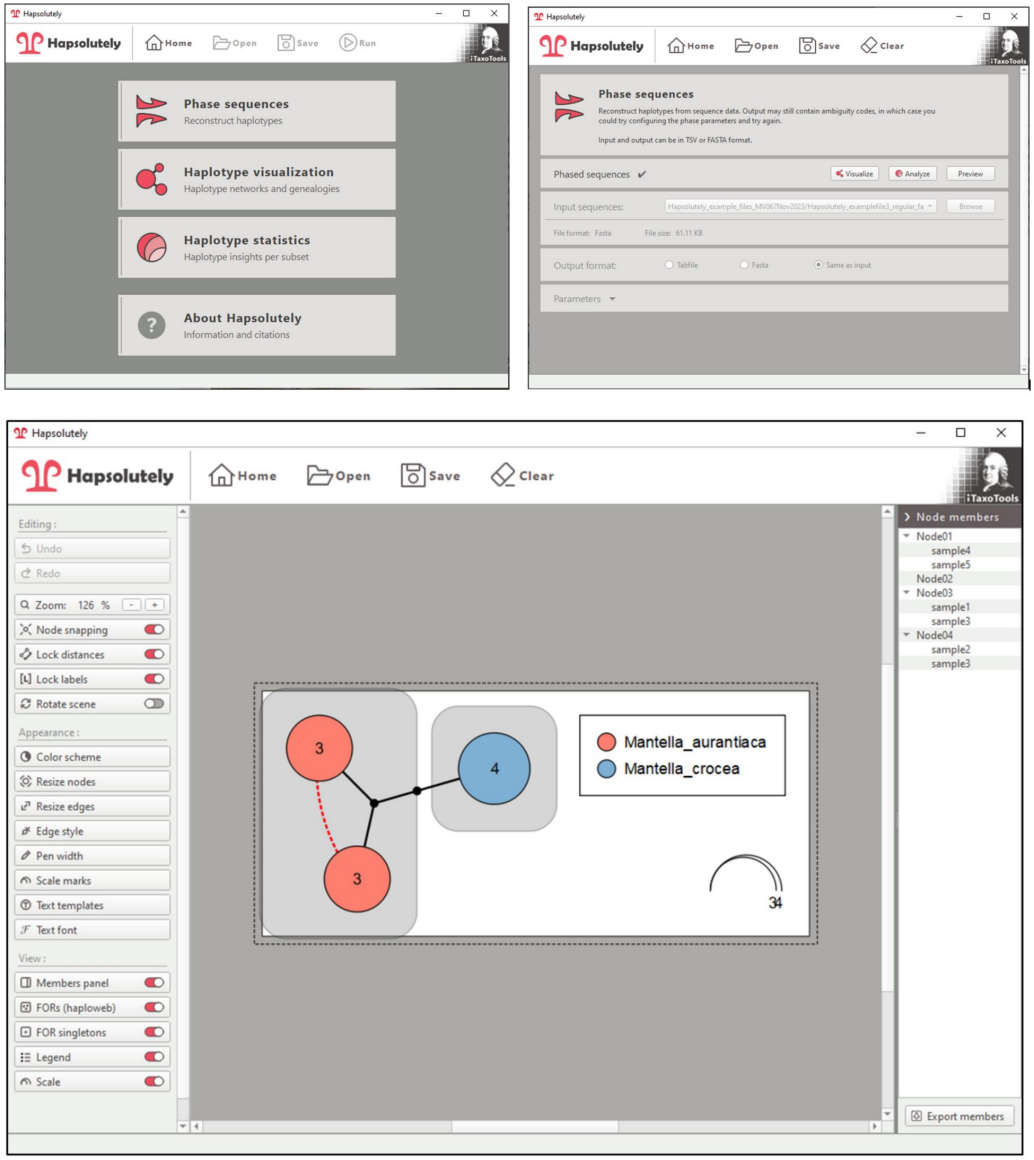
Screenshots of the Hapsolutely GUI. The images show the starting screen (upper left), ConvPhase module after completion of phasing (upper right), and network editor (below) of Hapsolutely, showing a simple example network with fields for recombination visualized as gray boxes and a dotted line indicating two haplotypes that are connected by one heterozygote individual. The task bar on the left allows adjusting the graph, the bar at the right lists which individuals belong to each node.

Finally, (iii) the reconstructed networks can be visualized and graphically adapted. The user can choose color scales and adjust every color manually, move nodes of the network, adapt annotations, select different representations of mutations separating alleles, and export publication-ready images in PNG, SVG, and PDF formats (Fig. 2).

Besides being the first tool to integrate phasing with the graphical reconstruction of haplotype networks and genealogies, Hapsolutely stands out by its focus on single-locus haplotypes, which remain important for species delimitation in an integrative taxonomy framework. For this purpose, it includes several features not readily available in other haplotype network editors: (i) visualization of haplowebs, which are a means to assess FFRs as a criterion for species delimitation (Doyle 1995, Flot *et al.* 2010), by adding additional curved connections between alleles that are shared in the same individual, and an underlying gray polygon marking all alleles per FFR; (ii) output of descriptive HS and FFR statistics as a YAML-compatible text file, which allows understanding whether individuals from primary species hypotheses share alleles or constitute separate FFRs, which again can be used as criteria to delimit species; and (iii) support for SPART and SPART-XML species partition files (Miralles *et al.* 2021), where users can choose consecutively the alternative species partitions included in these files, and assign colors in the network accordingly. The reliability of molecular-only species delimitation, especially when based on single or few markers, is highly dependent on the organismal and geographical context, and should be embedded in integrative approaches that take into account as many lines of evidence as possible (Ahrens 2024, Miralles *et al.* 2024). Hapsolutely facilitates the exploration of molecular differentiation across species partitions but is not a species delimitation tool *per se*. The program can, however, be helpful to inspect and visualize concordant differentiation of lineages across markers or discordance based, for instance, on incomplete lineage sorting. Users will need to keep in mind limitations due to sample bias. Hapsolutely combines original code written in C++ (Phase and several haplotype reconstruction algorithms from PopART), Haxe (SeqPHASE), and Python (Fitchi), with new code written primarily in Python. PySide6 was used for the GUI, BioPython for the construction of neighbor-joining trees, and the NetworkX package (Hagberg *et al.* 2008) for generating the initial graph layouts.

Phase (Stephens *et al.* 2001) as well as several network reconstruction algorithms from PopART (Leigh and Bryant 2015) were wrapped in the form of a CPython extension module. Both tools are available as installable Setuptools packages and expose their functionality through simple Python APIs. Standalone executables for Windows and Apple Macintosh (running both with Intel and Apple silicon processors) have been produced using PyInstaller. Import and export of SPART and SPART-XML format are carried out with a specifically developed module called SpartParser. The backend uses an extensible modular design in which configurable protocols are defined for reading/writing each file format and feeding to/from a standardized stream of markers. This consolidates the inherently different formats and allows for data analysis and manipulation.

Hapsolutely’s wrapped legacy code for phasing and haplotype networks or haplotype genealogy reconstruction is not designed for analysis of massive amounts of data but can easily handle single-locus alignments of 500–1000 bp and several hundred sequences. The TCS algorithm processes datasets of over 20 000 sequences and 50 alleles in <2 s on a personal computer. Phasing of the two example files provided with the program, containing 66 and 101 sequences of 732 and 451 bp in length, respectively, require 12 and 105 s for phasing, <1 s for haplotype generation, and 92 and 112 s for generation of Fitch genealogies (where inference of the maximum parsimony tree is the most time-consuming step).

We envisage future distributions of Hapsolutely to include improved functions for exploration of species partitions (see Miralles *et al.* 2021) and the option to compare the sharing of FFR among alternative species partitions and outputting this concordance information back into a SPART-XML file.

## Data Availability

The data underlying this article are available in the following repositories: The source code is openly available (GPL 3.0 license) on the GitHub repository (https://github.com/iTaxoTools/Hapsolutely). Compiled standalone executables of Hapsolutely and Convphase for MS Windows and Mac OS along with a detailed manual are available under from Github under https://github.com/iTaxoTools/Hapsolutely and https://github.com/iTaxoTools/ConvPhaseGui, as well as from https://www.itaxotools.org.
